# Supplementary material for: Rapid turnover of life-cycle-related genes in the brown algae
Source: Genome Biol. 2019 Feb 14;20:35. doi: 10.1186/s13059-019-1630-6 (PMC6374913; doi:10.1186/s13059-019-1630-6)
Supplement: Supplementary file 3 — Protein alignments, corresponding Gblocks html file and CDS sequences for the lineage conserved gametophyte-biased genes. (ZIP 2109 kb) [file 13059_2019_1630_MOESM3_ESM.zip › Con_GA_alignments/Ec-16_003980.aa_ali.fasta-gb.htm]

Ec-16\_003980.aa.fasta.trans.aa\_ali.fasta


## Gblocks 0.91b Results

Processed file: **Ec-16\_003980.aa.fasta.trans.aa\_ali.fasta**  
Number of sequences: **2**  
Alignment assumed to be: **Protein**  
New number of positions: **530** (selected positions are underlined in blue)

```
                         10        20        30        40        50        60
                 =========+=========+=========+=========+=========+=========+
Ec-16_003980     MKMKS---------NWVSTKTMLLLLVCLIP-CR-------LSSVVLVSAASASTASWTT
MSTRG.52918.1    MRLSESCAGQVPTNNMRATQRLLPLLCLLCPPCRPPMARPAWALLTMMASASAATASWKT
                               #################                   ##########


                         70        80        90       100       110       120
                 =========+=========+=========+=========+=========+=========+
Ec-16_003980     VWVDEFQGEDVDVDKWEFPDSTEEESDPVTV-----------EGGVRREQSDNNTSATEN
MSTRG.52918.1    VWVDEFESEDVDLEKWAFPAGNDDEAGAFAAGAGAEGAGDGRDGGIMNGDGFNKSTRTKE
                 #########################                  #################


                        130       140       150       160       170       180
                 =========+=========+=========+=========+=========+=========+
Ec-16_003980     HHRMR-SFSNPHLQAYIGADDYLVMVAAAAPPPQNSRGKGNGAEVLVGPGPSKP-PPRGR
MSTRG.52918.1    EHPARSALSNAYVQAYIGAHDYLVIASVTARSQSTNLNANDVAEASIQGASTLPTIPRGR
                 #####   ######################                          ####


                        190       200       210       220       230       240
                 =========+=========+=========+=========+=========+=========+
Ec-16_003980     IGTAHSANFVHGRVEISAKVSSRGSSGFWLMPSDAGTVGHTPCARVAIAEIRARQYHAAD
MSTRG.52918.1    ISSSHSADFSYGRVEIRAKLASRGSSGLWLMPSDVPVFGHTPCARISVAEIRAKKHNTTQ
                 #####################################################       


                        250       260       270       280       290       300
                 =========+=========+=========+=========+=========+=========+
Ec-16_003980     KSGPDITASLGFGDYEEDRWGCTSSSSWPDKGGGCSLPAEHGVDWSDGFHVYALEWEEDE
MSTRG.52918.1    NPARPLTASLSFGHYQEDRWGCTNSDVWPDEGSGCSSSAEHAVDWSDAFHVYALEWEEDE
                       ######################################################


                        310       320       330       340       350       360
                 =========+=========+=========+=========+=========+=========+
Ec-16_003980     IRWYVDGRQYCKKSAWFAKYPDDFPAPFNKAFHLEMGVAVGGGGDVGDGDSAEEEDPSSA
MSTRG.52918.1    IRWYVDGMQYCSKSSWFSKNPDDFPAPFNNAFHIEMGVETDDR-DSGSFSGADDGGDLSV
                 ######################################      ################


                        370       380       390       400       410       420
                 =========+=========+=========+=========+=========+=========+
Ec-16_003980     STRALHSPSVPTEMLVDYVRITQGPDGQWVP--PLESIA----ADGGLQPVALDGVTRSS
MSTRG.52918.1    LDRALHSPSELSEMLVDYVRITQGPQGVWAPAPPLEPLPADAAAEQGLGPLADGGLTSSP
                 ###############################            #################


                        430       440       450       460       470       480
                 =========+=========+=========+=========+=========+=========+
Ec-16_003980     PRMLDVIINLLAVSACVLGPYMAMTGLSRPRTNTILMGMEIGAFKAANASYLISGSYVMA
MSTRG.52918.1    PRALDIIINAIAVCACVLGPYMAMTGLSRARVNTIMMGMEIGAFKAANASYIFSGSYVMA
                 ############################################################


                        490       500       510       520       530       540
                 =========+=========+=========+=========+=========+=========+
Ec-16_003980     FVGAVTFGMLVGYLALHHRAMARWNTVAGLAVPALFVLIDIGMISDIGSRYVCWAIFFVL
MSTRG.52918.1    FAGAVTFGILCGFLAVRHRAMARWNTVAGLAVPALFVLIDVGMISDIGSKYVCWAIFFVL
                 ############################################################


                        550       560       570       580       590       600
                 =========+=========+=========+=========+=========+=========+
Ec-16_003980     TVTFAVLVFPSEHHAKGFVVATAATGSCVLKLGLGRLLGESGEIFKILDDPLGDWCHVSR
MSTRG.52918.1    TVTFALLVFPSEHHALGFAVAAAATGSCVLKLGLGWLLGESGEIFKILDDPIGDQCHASR
                 ############################################################


                        610       620       630       640       650       660
                 =========+=========+=========+=========+=========+=========+
Ec-16_003980     ICQSLIYVMGAMFFIGLSSQRCLRPLSKDVNNDGVTTSLEKQRSALFTPPRARSPLGASS
MSTRG.52918.1    VCQSLNFVMGGMFLIGLCSQKCMGPLSKDCG-SCIVADPDKE-SGLFTPPRYRFP-----
                 #############################              ############     


                        670       680       690       700       710       720
                 =========+=========+=========+=========+=========+=========+
Ec-16_003980     ARSSTHTLTRQDTCSTSSDFEIDDNQEASAGGQRQSGGDGGDKAAGRRQGNEKKGFRDYD
MSTRG.52918.1    ------------------------------------------------------------
                                                                             


                        730       740       750       760       770       780
                 =========+=========+=========+=========+=========+=========+
Ec-16_003980     EDEAFILGIGARRGRAPGAWERVSAGTRSFLQRVLRPFSSFRRHEQAPVSVTPDSPTPVP
MSTRG.52918.1    ------------------------------------------------------------
                                                                             


                        790       800       810       820       830       840
                 =========+=========+=========+=========+=========+=========+
Ec-16_003980     MPLTGVDSNAVVSSEGDTIELVIHSPHKRALRPHRPSGWFGSGGSSGGNSGGGGSDHRQS
MSTRG.52918.1    ------------------------------------------------------------
                                                                             


                        850       860       870       880       890       900
                 =========+=========+=========+=========+=========+=========+
Ec-16_003980     MPQFCLRDGQDGGNDRGLSSPSTNDYEDALSILSASELVTPDAEVPAAIAPAVASAGSAN
MSTRG.52918.1    ------------------------------------------------------------
                                                                             


                        910       920       930       940       950       960
                 =========+=========+=========+=========+=========+=========+
Ec-16_003980     TEGARSGRLADAFSEKWGSWVQDVNSSDSNDDGSGEGRRDDGRDGSGYRLAFQDNGEEKS
MSTRG.52918.1    ------------------------------------------------------------
                                                                             


                        970       980       990      1000      1010
                 =========+=========+=========+=========+=========+=========
Ec-16_003980     TAAGGSGGGGGGGCRSAREFDNEVLSPVPTEPRRRPAVLQRRRRRRRRCRGCGRNGTRQ
MSTRG.52918.1    -----------------------------------------------------------
```

```
Parameters used
Minimum Number Of Sequences For A Conserved Position: 2
Minimum Number Of Sequences For A Flanking Position: 2
Maximum Number Of Contiguous Nonconserved Positions: 8
Minimum Length Of A Block: 10
Allowed Gap Positions: None
Use Similarity Matrices: Yes
```

```
Flank positions of the 9 selected block(s)
Flanks: [15  31]  [51  85]  [104  125]  [129  150]  [177  233]  [247  338]  [345  391]  [404  629]  [644  655]  

New number of positions in Ec-16_003980.aa.fasta.trans.aa_ali.fasta-gb:  530  (52% of the original 1019 positions)
```
